# Pathological neutrophil extracellular traps hinder postoperative anal fistula wound healing and are attenuated by Zuoqing granule via suppression of the Nox4 pathway

**DOI:** 10.3389/fimmu.2025.1730184

**Published:** 2026-01-20

**Authors:** Xiaoli Fang, Heng Deng, Ming Li, Xiang Gao, Chunrong He, Hui Liu

**Affiliations:** 1 Department of Anorectal Surgery, The First Affiliated Hospital of Anhui University of Chinese Medicine, Hefei, China; 2 Department of Anorectal Surgery, Second Affiliated Hospital, Anhui University of Chinese Medicine, Hefei, China; 3 Department of Anorectal Surgery, The Third Affiliated Hospital of Anhui University of Chinese Medicine, Hefei, China; 4 General Practice Department, Hefei Haiheng Health Service Center, Hefei, China; 5 General Practice Department, Anhui Provincial Second People’s Hospital, Hefei, Anhui, China

**Keywords:** anal fistula, chronic inflammation, NEtosis, NOX4, wound healing, Zuoqing granule

## Abstract

**Background:**

The impaired healing of postoperative anal fistula wounds often complicated by a contaminated environment and persistent inflammation. The key pathological immune events that sustain this chronic inflammatory milieu remain largely unknown. We hypothesized that dysregulated neutrophil extracellular trap (NET) formation (NETosis)—a potent driver of tissue damage—might be a pathological feature and potential therapeutic target.

**Methods:**

Using a rat model of contaminated wounds akin to postoperative anal fistula, we characterized NETosis via immunofluorescence (CitH3/CD66b), transmission electron microscopy, and ELISA. The involvement of the Nox4/ROS/PI3K/Akt/PADI4 pathway was assessed. The therapeutic potential of Zuoqing Granule (ZQG), a clinically used traditional Chinese formulation, was evaluated both *in vivo* and in PMA-stimulated rat neutrophils *in vitro*. Bacterial burden were also assessed.

**Results:**

We identified pervasive NETosis as a pathological hallmark of non-healing anal fistula wounds, accompanied by a surge in pro-inflammatory cytokines (IL-2, IL-5, IL-6, IL-12, TNF-α) and a ~15.5-fold increase in bacterial load compared to controls. ZQG treatment dose-dependently accelerated wound closure, resolved inflammation, reduced bacterial burden, and suppressed NETosis by up to 75.1% at day 7. Mechanistically, ZQG inhibited the Nox4/ROS/PI3K/Akt/PADI4 axis. *In vitro*, ZQG reduced PMA-induced NETosis by 63.0% and superoxide production by 58.1%, comparable to Nox4 knockdown.

**Conclusion:**

Our study establishes aberrant NETosis as a pathological feature and potential therapeutic target in anal fistula-like wounds. We further identify ZQG as a promising candidate therapy that alleviates this pathology by suppressing the Nox4/ROS/PI3K/Akt/PADI4 pathway, without compromising bacterial clearance.

## 1 Introduction

Anal fistula, a common sequelae of anorectal abscesses, presents a significant surgical challenge due to high recurrence rates and prolonged postoperative healing times (1, 2). A critical yet under-investigated feature in the management of anal fistula is the persistent, low-grade inflammation that characterizes the non-healing postoperative wound bed, preventing tissue regeneration (3). The underlying mechanisms sustaining this chronic inflammatory state are not fully elucidated, leading to a lack of targeted therapies.

Neutrophils are the first responders to surgical trauma and infection (4). Beyond phagocytosis, their activated form of cell death, NETosis, results in the release of Neutrophil Extracellular Traps (NETs) (5). While NETs are crucial for pathogen entrapment, excessive or dysregulated NETosis is increasingly recognized as a key contributor to a range of chronic inflammatory and autoimmune diseases by damaging tissues and perpetuating immune activation (6, 7). Notably, NETs have been shown to impair healing in diabetic wounds and burns (8, 9). However, the presence and pathological significance of NETs in the specific context of anal fistula postoperative wounds remain completely unknown. We therefore hypothesized that persistent, dysregulated NETosis constitutes a fundamental mechanism sustaining the chronic inflammation and impairing healing in postoperative anal fistula wounds.

Zuoqing Granule (ZQG) is a traditional Chinese medicine formula. It is composed of *Indigo Naturalis*, *Cortex Phellodendri*, *Radix Sophorae Flavescentis*, *Herba Agrimoniae*, and *Radix Sanguisorbae*. Our prior clinical practice and research have consistently demonstrated its efficacy in reducing inflammation, edema, and discharge (10). Network pharmacology analyses suggest it may target inflammatory pathways (11). Nevertheless, its potential interaction with neutrophil-driven inflammation and NETosis has never been investigated.

Therefore, this study was designed to address two fundamental questions: Is aberrant NETosis a prominent pathological feature and potential contributor to non-healing in anal fistula wounds? If so, can the therapeutic efficacy of ZQG be linked to its ability to modulate this novel pathway? In this study, we employed an integrated *in vivo* and *in vitro* approach to investigate the presence and role of NETs in a model of anal fistula-like wounds, and to evaluate whether the therapeutic effect of ZQG is associated with the modulation of NETosis and its regulatory pathways.

## 2 Materials and methods

### 2.1 Preparation and quality control of ZQG

ZQG, a standardized clinical preparation, was provided by Huarun Sanjiu Pharmaceutical Co., Ltd. (Hefei, China; Manufacturing License: Z2024432, Batch No. 20240654). Each gram of granules is equivalent to 3.0 grams of crude drugs. The formula is composed of *Indigo Naturalis* (3 g, a natural pigment derived from the leaves and stems of *Polygonum tinctorium Aiton*), the root bark of *Phellodendron chinense C.K.Schneid.* (6 g), the roots of *Sophora flavescens Aiton* (6 g), the aerial parts of *Agrimonia pilosa Ledeb.* (6 g), and the roots of *Sanguisorba officinalis L.* (6 g). The botanical origins of all crude drugs were authenticated by the manufacturer’s quality control department according to the Chinese Pharmacopoeia (2020 edition). Documentation of species identification (Supplier’s Certificate of Analysis for Batch No. 20240654) is kept on file by the manufacturer and available upon request. Quality control and chemical profiling of ZQG, including the identification of active compounds such as matrine, oxymatrine, and berberine, were performed using Ultra-Performance Liquid Chromatography-Mass Spectrometry (UPLC-MS) as previously described (11). The UPLC-MS chromatogram is provided in the Supplementary Material (**Supplementary Figure S1**), and the corresponding peaks for key compounds are listed in **Supplementary Table S1**.

For topical application, ZQG granules were dissolved in sterile physiological saline (0.9% NaCl) to prepare three treatment solutions:

Low-dose (ZQG-L): 0.5 g granules/mL (equivalent to 1.5 g crude drugs/mL)

Medium-dose (ZQG-M): 0.75 g granules/mL (equivalent to 2.25 g crude drugs/mL)

High-dose (ZQG-H): 1.0 g granules/mL (equivalent to 3.0 g crude drugs/mL)

These concentrations were designed to deliver daily doses of approximately 5, 7.5, and 10 g of granules per kg body weight, respectively, based on a standardized application volume of 1 mL per rat.

### 2.2 Animals and ethical statement

Male Sprague-Dawley rats (180–220 g) were purchased from Beijing Vital River Laboratory Animal Technology Co., Ltd. (Beijing, China). Animals were housed under standard conditions (12-h light/dark cycle, 22 ± 2°C, 50–60% humidity) with free access to food and water. All animal experimental procedures were approved by the Ethics Committee of First Affiliated Hospital of Anhui University of Chinese Medicine (Approval No: 2025AH-81-01) and were conducted in strict accordance with the NIH Guide for the Care and Use of Laboratory Animals and the ARRIVE guidelines (12).

### 2.3 Postoperative anal fistula wound model establishment and experimental design

Rats were anesthetized with an intraperitoneal injection of 3% pentobarbital sodium (40 mg/kg). The dorsal region was shaved and disinfected. A full-thickness circular skin defect (approx. 19 mm in diameter) was created by surgical excision down to the fascial layer. To simulate the contaminated environment of an postoperative anal fistula wound, 0.5 mL of fecal supernatant (prepared from fresh rat stool suspended in saline and centrifuged at 3,000 × g for 10 min) was applied daily to the wound surface before dressing change (13), and this application was maintained daily throughout the 7-day experimental period.

Rats were randomly assigned to five groups (n=10 per group): Control, Model, ZQG-L, ZQG-M, and ZQG-H. The Control group underwent surgery without fecal supernatant application and received saline-saturated gauze. The Model group received the full surgical and fecal application procedure with saline-saturated gauze. The ZQG-L, ZQG-M, and ZQG-H groups were model rats treated topically with gauze pads saturated with 1 mL of low-, medium-, or high-dose ZQG solution, respectively.

### 2.4 Treatment protocol

Freshly prepared ZQG or saline solutions were applied daily after wound cleansing for 7 days. A sterile gauze pad (2 cm × 2 cm) was saturated with the respective solution and placed directly onto the wound surface, which was then secured with a transparent film dressing (Tegaderm™) to ensure adequate contact and prevent leakage.

### 2.5 Wound assessment and monitoring

**Figure 1:**
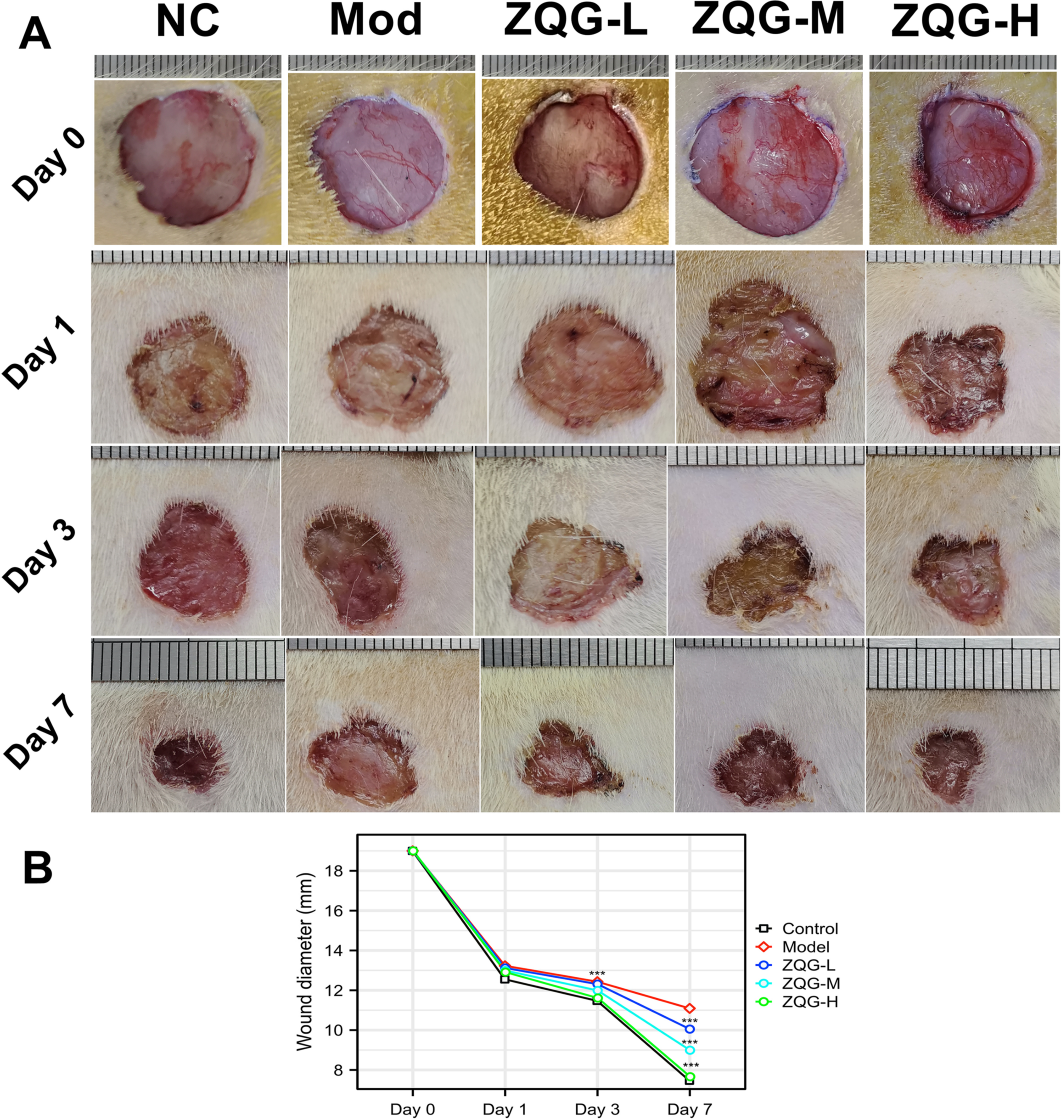
Macroscopic wound healing. **(A)** Representative macroscopic images of wounds on days 0, 1, 3, and 7. **(B)** Quantification of wound diameter. ***p < 0.001 vs. Model group.

The diameter of the wound was measured in the same direction on the first, third, and seventh days after the operation. A standard ruler was placed adjacent to the wound as a scale reference during photographic documentation (as shown in **Figure 1A**), and the wound diameter was determined based on these calibrated images.

### 2.6 Sample collection

On day 7 post-operation, rats were euthanized by an intraperitoneal injection of an overdose of sodium pentobarbital (150 mg/kg). Wound tissues were then harvested. A portion was fixed in 4% paraformaldehyde for histology and immunofluorescence. Another portion was snap-frozen in liquid nitrogen and stored at -80°C for subsequent protein, RNA, and cytokine analysis. For TEM, 1 mm³ tissue pieces were fixed in 2.5% glutaraldehyde overnight.

### 2.7 Histological analysis

Paraffin-embedded tissues were sectioned (4 μm) and stained with Hematoxylin and Eosin (H&E) for general morphological assessment. The histological score was evaluated in a blinded manner by two independent pathologists according to modified criteria based on previous studies (14, 15). The scoring system assessed the following parameters: (i) degree of inflammatory cell infiltration, (ii) formation and maturity of granulation tissue, (iii) extent of re-epithelialization, and (iv) restoration of tissue architecture. Each parameter was scored on a scale of 0 (normal) to 3 (most severe), and the scores were summed to generate a total histological score for each sample.

### 2.8 Enzyme-linked immunosorbent assay

Frozen wound tissues were homogenized in RIPA buffer with protease inhibitors. The homogenates were centrifuged at 12,000×g for 15 min at 4°C, and the supernatants were collected. The concentrations of interleukin-2 (IL-2), interleukin-5 (IL-5), interleukin-6 (IL-6), interleukin-12 (IL-12), and tumor necrosis factor-alpha (TNF-α) in the supernatants were quantified using specific commercial rat ELISA kits (R&D Systems, Catalog # R6000B) according to the manufacturers’ instructions. The total protein concentration of each sample was determined by a BCA assay kit for normalization. Results are expressed as pg of cytokine per mg of total protein.

### 2.9 Bacterial load quantification

The bacterial burden in wound tissues was determined by counting colony forming units (CFU) at the endpoint (day 7). Approximately 50 mg of snap-frozen tissue was homogenized in 1 mL of sterile phosphate-buffered saline (PBS, Sigma-Aldrich, P4417). The homogenate was subjected to 10-fold serial dilutions, and 100 μL of each dilution was plated on LB agar plates (BD Biosciences, 244520). After 24 h of incubation at 37°C, colonies were counted and normalized to tissue weight (CFU/g).

### 2.10 Immunofluorescence staining

Paraffin sections were deparaffinized, rehydrated, and subjected to antigen retrieval. After blocking with 5% BSA, sections were incubated overnight at 4°C with primary antibodies: rabbit anti-Citrullinated Histone H3 (CitH3, 1:200, Abcam, ab5103) and mouse anti-CD66b (1:100, BD Biosciences, 551481). After washing, sections were incubated with corresponding fluorescent secondary antibodies (Alexa Fluor 488 and 594, 1:500, Invitrogen) for 1 h at room temperature. Intracellular reactive oxygen species (ROS) detection: tissue sections were incubated with Dihydroethidium (5 μM, Beyotime, S0063) in a dark, humidified chamber at 37°C for 30 min. Nuclei were counterstained with DAPI. Images were captured using a confocal microscope (Nikon A1 or Olympus FV3000). The number of CitH3^+^CD66b^+^ double-positive cells per high-power field (HPF, 400x) was counted to quantify NETosing neutrophils.

### 2.11 Transmission electron microscopy

The glutaraldehyde-fixed tissue samples were post-fixed in 1% osmium tetroxide, dehydrated in a graded ethanol series, and embedded in epoxy resin. Ultrathin sections (70 nm) were stained with 2% uranyl acetate and lead citrate. The ultrastructural morphology of neutrophils was observed and imaged using a Hitachi HT-7800 transmission electron microscope to identify classic features of NETosis.

### 2.12 Western blot analysis

Total protein was extracted from wound tissues. Equal amounts of protein (30 μg) were separated by 10% SDS-PAGE and transferred to PVDF membranes. After blocking, membranes were incubated overnight at 4°C with primary antibodies against: Nox4 (1:1000, Proteintech, 14347-1-AP), PI3K (1:1000, CST, #4257), p-Akt (Ser473, 1:2000, CST, #4060), Akt (1:1000, CST, #4691), PADI4 (1:1000, Abcam, ab214810), and β-Actin (1:5000, Proteintech, 60008-1-Ig). After incubation with HRP-conjugated secondary antibodies, protein bands were visualized using an ECL detection system and quantified using ImageJ software.

### 2.13 Quantitative real-time PCR

Total RNA was extracted from wound tissues using TRIzol Reagent (Invitrogen). cDNA was synthesized using a PrimeScript RT Kit (Takara). qPCR was performed in triplicate using TB Green Premix (Takara) on a QuantStudio 5 system (Applied Biosystems). Gene expression was quantified using the 2^(-ΔΔCt) method with β-actin as the endogenous control. The primer sequences used are listed in **Table 1**.

**Table 1:** qPCR primer sequences.

Gene	Accession no.	Primer sequence (5’ to 3’)	Product size
Nox4	NM_053524.1	F: GACAAGAAGGAGATTGGCGT R: GATGAAGGCGAGTTGAAGCC	112 bp
PI3K*-*p85α	NM_001024698.1	F: CAGAGGATGCTGGCTTTGAC R: CAGGCGTTTTGTAACCAGGA	150 bp
Akt1	NM_033230.2	F: GAGCGACGTGGCTATTGTGA R: GATGAGGTCGTGCATGAGGT	98 bp
PADI4	NM_001106847.1	F: TGGCAACCTCTGTGTCATCG R: AGGCGTAGTTGTAGCCGTTC	105 bp
β-actin	NM_031144.3	F: CCCATCTATGAGGGTTACGC R: TTTAATGTCACGCACGATTTC	150 bp

### 2.14 *In vitro* NETosis induction and intervention

#### 2.14.1 Cell culture and viability assessment

Rat peripheral blood neutrophils (ZenBio, Inc., Cat# SER-NHPPB-25M) were cultured to establish an *in vitro* model. Briefly, cryopreserved neutrophils were rapidly thawed and seeded in RPMI-1640 medium supplemented with 10% fetal bovine serum (FBS) at a density of 1×10^6^ cells/well (6-well plate) and maintained at 37°C under 5% CO_2_. Cell viability after thawing was consistently >90% as assessed by Trypan Blue exclusion, and only batches meeting this criterion were used for experiments.

Prior to functional assays, the potential cytotoxicity of ZQG was evaluated. Neutrophils were treated with a gradient concentration of ZQG (0, 0.25, 0.5, 1.0, and 2.0 mg/mL) for 6 h. Cell viability was then assessed using a Cell Counting Kit-8 (CCK-8; Dojindo, Japan) according to the manufacturer’s instructions. After a 2-h incubation with the CCK-8 reagent, the absorbance was measured at 450 nm using a microplate reader. Based on the results, a non-cytotoxic concentration of 1 mg/mL was selected for all subsequent experiments.

#### 2.14.2 Gene knockdown via siRNA transfection

To genetically inhibit Nox4 expression, neutrophils were transfected with 20 nM ON-TARGETplus SMARTpool siRNA targeting rat Nox4 (Dharmacon) or a non-targeting control siRNA (Dharmacon) using Lipofectamine RNAiMAX transfection reagent, strictly following the manufacturer’s protocol. Following a 48-h transfection period to allow for maximal protein knockdown, cells were subjected to subsequent treatments. Knockdown efficiency was confirmed by Western blot analysis.

#### 2.14.3 Drug treatment and NETosis induction

NETosis was pharmacologically induced using phorbol 12-myristate 13-acetate (PMA). A 1 mM PMA stock solution was prepared in DMSO and diluted in culture medium to a final working concentration of 100 nM. ZQG was prepared as a 10 mg/mL stock solution in sterile saline, filter-sterilized (0.22 μm), and diluted to its final working concentration of 1 mg/mL in culture medium.

Neutrophils were divided into the following experimental groups:

Control: Treated with complete culture medium only.

PMA: Stimulated with 100 nM PMA for 4 h to induce NETosis.

PMA + ZQG: Pre-treated with 1 mg/mL ZQG for 2 h, followed by co-stimulation with 100 nM PMA for 4 h.

PMA + si-Nox4: Neutrophils transfected with Nox4-targeting siRNA for 48 h, followed by stimulation with 100 nM PMA for 4 h.

### 2.15 Statistical analysis

Data are presented as mean ± standard error of the mean (SEM). Statistical analysis was performed using GraphPad Prism 9.0 software. One-way analysis of variance (ANOVA) followed by Tukey’s *post hoc* test was used for comparisons among multiple groups. A value of p < 0.05 was considered statistically significant.

## 3 Results

### 3.1 Postoperative anal fistula wounds exhibit impaired healing and sustained inflammation that is rescued by ZQG

Macroscopic observation confirmed the successful establishment of the impaired healing model. The wounds in the Model group showed significantly delayed closure compared to the Control group from day 3 onwards (**Figures 1A, B**).

**Figure 2:**
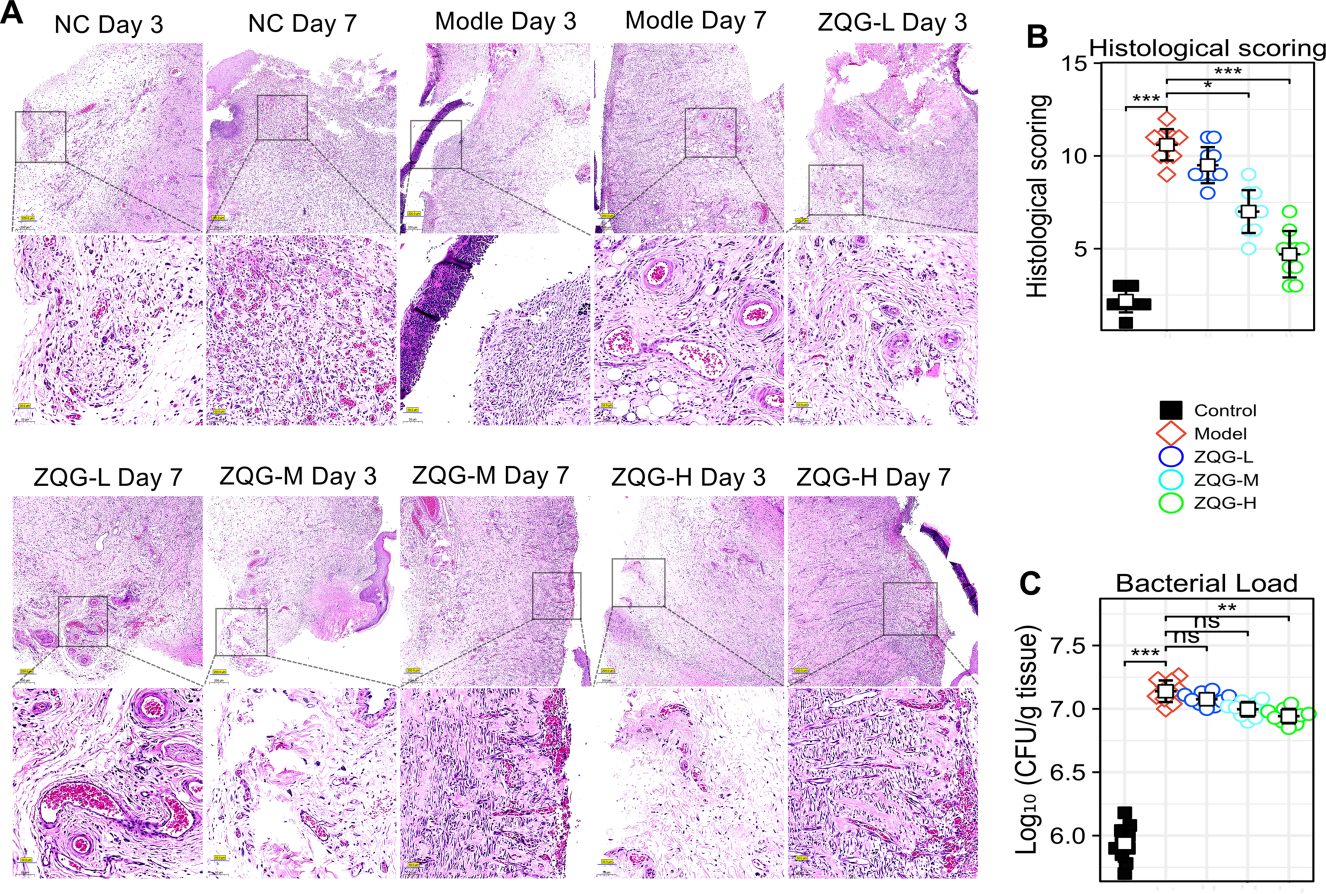
Histopathological analysis, scoring, and bacterial load in wound tissues. **(A)** Representative H&E-stained sections of wound tissues on day3, and 7. **(B)** Histological scoring. **(C)** Quantitative analysis of bacterial burden (Log_10_ CFU/g tissues). *p < 0.05, **p < 0.01, ***p < 0.001 vs. Model group.

Consistent with the macroscopic findings, histological analysis revealed severe inflammation, tissue damage, and immature granulation tissue in the Model group, as evidenced by a significantly higher histological score (10.6 ± 0.84) compared to the Control group (2.2 ± 0.63; p < 0.001) (**Figures 2A, B**). Furthermore, ELISA demonstrated a significant upregulation of IL-2, IL-5, IL-6, IL-12, and TNF-α in the Model group compared to the Control group (p < 0.001 for all cytokines; **Table 2**).

**Table 2:** Effects of ZQG on inflammatory cytokine levels.

Cytokine	Control	Model	ZQG-L	ZQG-M	ZQG-H
IL-2	29.36 ± 2.45	124.90 ± 4.73	83.24 ± 3.67 ***	59.18 ± 3.27***	45.37 ± 2.38***
IL-5	12.06 ± 1.12	34.47 ± 1.87	27.43 ± 1.56***	19.79 ± 1.32***	14.82 ± 0.98***
IL-6	82.46 ± 2.87	173.92 ± 4.12	137.36 ± 3.45***	109.35 ± 3.12***	92.52 ± 2.76***
IL-12	25.27 ± 1.23	64.26 ± 2.34	49.12 ± 1.87***	38.21 ± 1.56***	29.87 ± 1.34***
TNF-α	39.08 ± 1.87	117.15 ± 3.45	83.37 ± 2.98***	63.61 ± 2.56***	49.48 ± 1.92***

Data are presented as mean ± SEM (n = 10). Asterisks indicate significant differences compared to the Model group as determined by Tukey’s *post hoc* test following one-way ANOVA: ***p < 0.001 vs. Model group.

Treatment with Zuoqing Granule (ZQG) effectively rescued this pathological phenotype. ZQG application, particularly at the high dose, dose-dependently accelerated wound closure (**Figures 1A, B**), ameliorated histopathological damage (**Figures 2A, B**), and suppressed the production of pro-inflammatory cytokines (**Table 2**).

Quantification of the bacterial load in wound tissues showed that the Model group exhibited a significantly higher bacterial burden compared to the Control group (p < 0.001) (**Figure 2C**). This load was significantly reduced by high-dose ZQG treatment (ZQG-H vs. Model, p < 0.05; **Figure 2C**).

### 3.2 Persistent NETosis is a hallmark of non-healing postoperative anal fistula wounds

**Figure 3:**
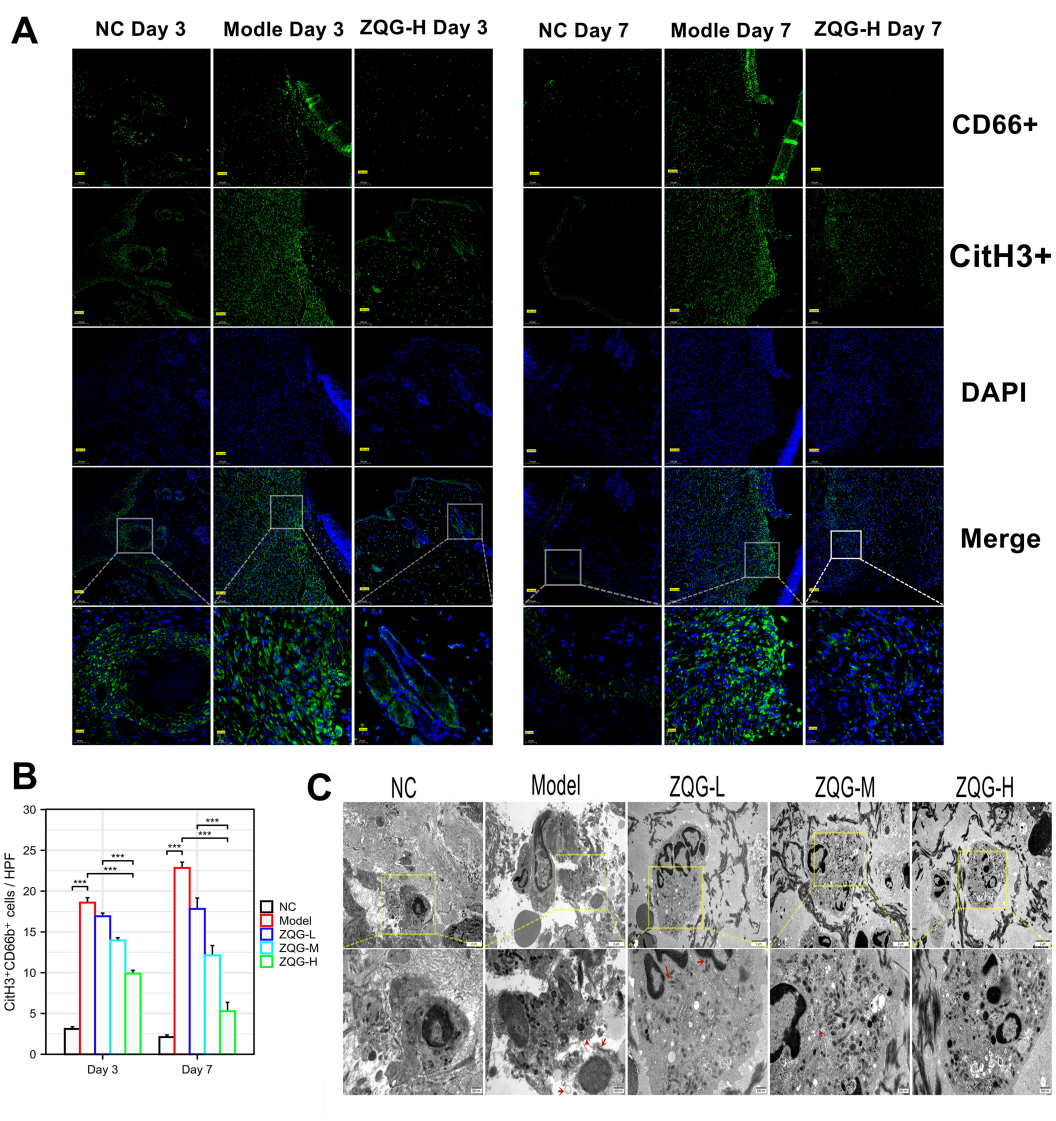
ZQG suppresses NETosis in postoperative anal fistula wounds. **(A)** Representative immunofluorescence images of day 3 and 7 wound sections stained for CitH3 (green, NET marker), CD66b (green, neutrophil marker), and DAPI (blue, nuclei). **(B)** Quantification of CitH3^+^CD66b^+^ cells per high-power field (HPF). **(C)** Representative transmission electron microscopy (TEM) images showing neutrophil ultrastructure. Red arrows indicate NET-like structures (decondensed chromatin and extruded filaments). Data are mean ± SEM; ***p < 0.001 vs. Model group.

Immunofluorescence co-staining for CitH3 and CD66b revealed a massive infiltration of NETosing neutrophils (CitH3^+^CD66b^+^ cells) in the wound bed of the Model group, which was minimal in Control animals (**Figures 3A, B**). Transmission electron microscopy (TEM) visually captured the classic ultrastructural features of NETosis, including neutrophil chromatin decondensation and the release of NET-like filaments, in the Model group (**Figure 3C**).

### 3.3 ZQG potently suppresses NETosis *in vivo*

ZQG treatment strikingly suppressed NETosis *in vivo*. Compared to the Model group, ZQG treatment dose-dependently reduced the number of CitH3^+^CD66b^+^ cells. Specifically, the ZQG-H group reduced the number of NETosing neutrophils by approximately 46.7% at day 3 (from 18.59 ± 0.18 to 9.90 ± 0.12 cells/HPF) and by 75.1% at day 7 (from 22.84 ± 0.20 to 5.69 ± 0.12 cells/HPF) (p < 0.001 for both) (**Figures 3A, B**). The near-complete absence of NETotic structures in the ZQG-H group, as confirmed by TEM, provided direct visual evidence of this inhibitory effect (**Figure 3C**).

### 3.4 ZQG suppresses the Nox4/ROS/PI3K/Akt/PADI4 pathway to inhibit NETosis

**Figure 4:**
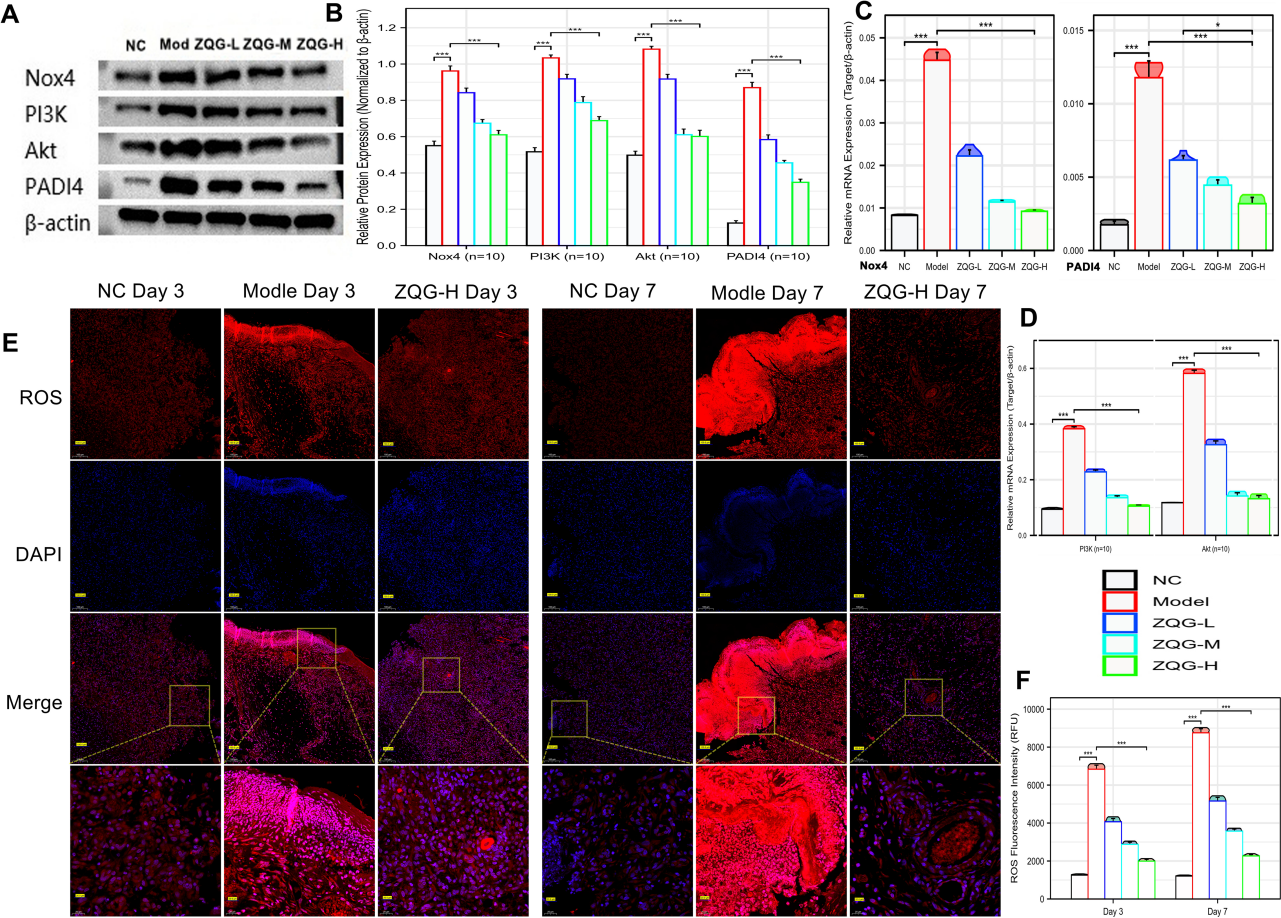
ZQG inhibits the Nox4/ROS/PI3K/Akt/PADI4 pathway in wound tissue. **(A)** Representative Western blot bands of Nox4, p-PI3K, p-Akt, Akt, and PADI4. **(B–D)** Densitometric quantification of protein expression **(B)** and qPCR analysis of mRNA expression **(C, D)**, normalized to β-actin. **(E)** Representative immunofluorescence images of intracellular ROS levels in wound granulation tissue (stained with DHE, red). Nuclei were counterstained with DAPI (blue). Scale bar, 100 μm. **(F)** Quantitative analysis of relative DHE fluorescence intensity. Data are presented as mean ± SEM (n = 10 fields of view). *p < 0.05, ***p < 0.001 vs. Model group.

Western blot and qPCR analyses revealed that the key mediators of NETosis were significantly upregulated in the Model group. The protein expression of Nox4, p-PI3K, p-Akt (Ser473), and PADI4 increased by approximately 1.8-, 2.1-, 2.3-, and 6.4-fold, respectively, compared to the Control group (all p < 0.001; **Figures 4A, B**). A concomitant surge in their mRNA levels was also confirmed (**Figures 4C, D**). Immunofluorescence analysis detected a 5.4-fold increase in intracellular ROS in the Model group (p < 0.001 vs. Control; **Figures 4E, F**).

ZQG treatment dose-dependently suppressed the activation of this entire signaling axis. The high dose (ZQG-H) was most effective, reducing the protein levels of Nox4, p-PI3K, p-Akt, and PADI4 to only 1.1-, 1.2-, 1.1-, and 2.6-fold of the Control group, respectively (all p < 0.001 vs. Model; **Figures 4A, B**). Accordingly, ZQG-H also attenuated the ROS fluorescence intensity to a level that was only 1.8-fold higher than the Control group (p < 0.001; **Figures 4E, F**).

### 3.5 ZQG directly inhibits the Nox4/ROS/NETosis/inflammation axis in rat neutrophils

**Figure 5:**
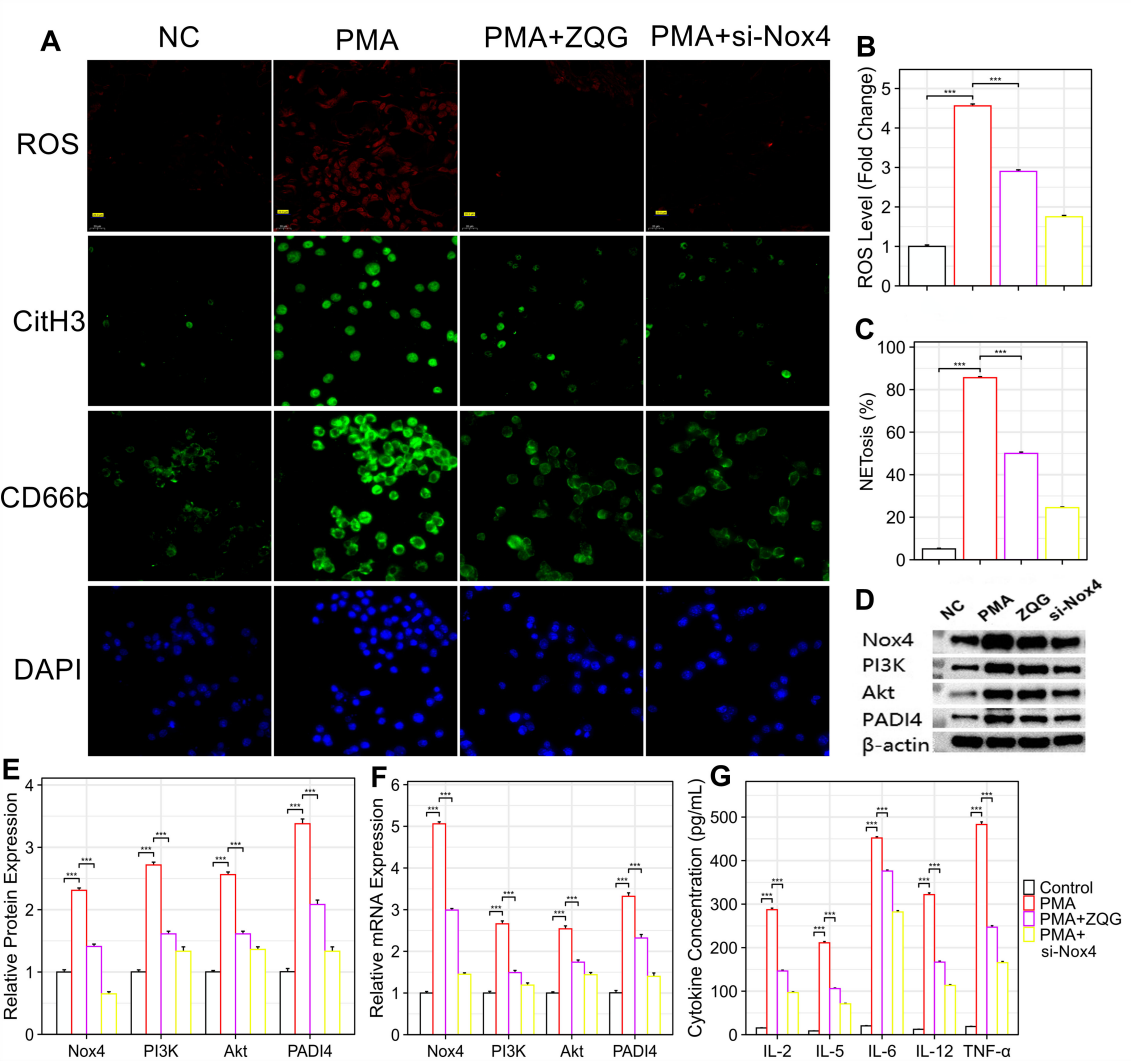
ZQG attenuates PMA-induced NETosis and inflammatory responses in human neutrophils via targeting the Nox4/ROS pathway. **(A)** ZQG suppresses oxidative burst and NET formation. Representative immunofluorescence images showing intracellular ROS (stained by DHE, red) and NETosis (marked by CitH3, green). Nuclei are counterstained with DAPI (blue). Scale bars: 20 μm. **(B)** Quantitative analysis of intracellular ROS levels. **(C)** Quantification of NETosis, expressed as the percentage of Sytox Green^+^ cells. **(D)** Western blot analysis demonstrating the effects of ZQG and si-Nox4 on the protein expression of Nox4, p-Akt, and PADI4. **(E)** Densitometric quantification of Western blot results. **(F)** qPCR analysis of Nox4, PI3K, Akt, and PADI4 mRNA expression. **(G)** ELISA analysis of pro-inflammatory cytokine levels (IL-2, IL-5, IL-6, IL-12, TNF-α) in cell culture supernatants. Data are presented as mean ± SEM (n=10 independent experiments). ***p < 0.001 vs. PMA group.

A CCK-8 assay confirmed that the concentration of ZQG (1 mg/mL) used was non-cytotoxic. PMA stimulation increased intracellular superoxide by 4.51 ± 0.12-fold compared to the Control. This was significantly reduced by ZQG pre-treatment (to 1.89 ± 0.08-fold, p < 0.001), while genetic knockdown of Nox4 demonstrated an even more potent inhibitory effect (to 1.72 ± 0.09-fold) (**Figures 5A, B**).

A parallel effect was observed on NETosis. The percentage of NETosing neutrophils (Sytox Green^+^/CitH3^+^) surged to 85.4 ± 3.2% upon PMA stimulation but was significantly reduced to 31.6 ± 2.8% by ZQG pre-treatment (p < 0.001). Si-Nox4 pre-treatment showed the strongest suppression, reducing NETosis to 26.3 ± 2.1% (**Figures 5A, C**).

Western blot and qPCR analyses demonstrated that PMA stimulation pronouncedly upregulated the protein and mRNA levels of Nox4, PI3K, Akt, and PADI4. These increases were significantly suppressed by both ZQG treatment and si-Nox4 transfection (**Figures 5D–F**).

ELISA analysis demonstrated that PMA stimulation significantly elevated the secretion of pro-inflammatory cytokines (IL-2, IL-5, IL-6, IL-12, and TNF-α), an effect that was significantly suppressed by both ZQG treatment and si-Nox4 pre-treatment (**Figure 5G**).

## 4 Discussion

This study delivers two pivotal advances. First, it identifies excessive and persistent NETs formation—NETosis—is a prominent pathological feature in a model of non-healing contaminated wounds akin to postoperative anal fistula. Second, it establishes the traditional Chinese formula ZQG, as an effective intervention that alleviates this pathology and accelerates healing, closely associated with the suppression of the Nox4/ROS/PI3K/Akt/PADI4 axis.

The failure of postoperative anal fistula wounds to heal is often attributed to persistent low-grade inflammation (16, 17), yet the underlying cellular effectors remain unclear. As essential for innate immunity (18), NETs can become detrimental when produced excessively or persistently (19, 20). Our findings demonstrate the sustained presence of NETs from day 3 to 7 coincided with the peak inflammatory phase and stalled healing, and their abundance correlated strongly with elevated cytokines and bacterial burden. Critically, pharmacological suppression of NETosis observed with ZQG was concurrently associated with improvement in all aspects of healing—from macroscopic closure and histology to inflammation and bacterial clearance. This convergence of evidence positions excessive NETosis as an integral, targetable component within the pathogenic cycle of non-healing wounds (21–23).

We identified the Nox4/ROS/PI3K/Akt/PADI4 pathway as a central signaling cascade associated with NETosis in anal fistula wounds. This finding is consistent with previous studies in other chronic inflammatory models (24), such as in lung ischemia/reperfusion injury (25, 26), leading to PADI4-mediated histone citrullination and NET release (27, 28). Similarly, in diabetes-Related Biofilm Infections and diabetic wound, aberrant NETosis driven by similar pathways has been linked to impaired healing (29, 30). This mechanistic link is supported by our complementary *in vivo* and *in vitro* models.

ZQG treatment dose-dependently accelerated wound closure, resolved inflammation, and inhibited NETosis. This effect was mechanistically associated with suppression of the Nox4/ROS/PI3K/Akt/PADI4 axis, as evidenced by consistent downregulation of Nox4 and its downstream effectors both *in vivo* and *in vitro* (31). Notably, the reduction in NETosis did not compromise bacterial clearance; instead, high-dose ZQG significantly lowered wound bacterial burden. This suggests that ZQG reprograms, rather than broadly suppresses, the neutrophil response, curbing pathogenic NETosis while maintaining antimicrobial capacity—a balance vital for transitioning from inflammation to healing. As a multi-component formulation, ZQG’s efficacy likely arises from synergistic actions of its constituents (e.g., antimicrobial berberine) that collectively target infection and dysregulated immunity (**Supplementary Figure S1**, **Supplementary Table S1**). Future studies to isolate the key NETosis-inhibiting component(s) are warranted.

Our study has several limitations. The dorsal contaminated wound model, while simulating key features of inflammation and bacterial burden, does not replicate the specific anatomy of an anal fistula. Another limitation is the absence of experiments designed to directly disrupt NETs (e.g., using DNase I or PAD4 inhibitors) to establish causality (23, 32). Such functional studies would be a crucial next step to definitively prove that NETosis is a driver, rather than merely a correlate, of the pathology. Future work employing these tools will be essential to validate NETosis as a direct therapeutic target in this context. Additionally, future studies utilizing specific Nox4 inhibitors or conditional knockout models are warranted to definitively establish Nox4 as the primary target of ZQG and to dissect its effects from other neutrophil functions.

## 5 Conclusion

In conclusion, our work redefines the pathophysiology of impaired healing in anal fistula-like wounds by highlighting the central role of NETosis. We identify ZQG as a promising, mechanism-based therapy that targets this process via the Nox4/ROS/PI3K/Akt/PADI4 pathway, without undermining fundamental host defense. These findings bridge clinical observation with molecular immunology, offering a novel therapeutic strategy and positioning NETosis as a central target for managing postoperative anal fistula and similar chronic inflammatory wounds.

## Data Availability

The original contributions presented in the study are included in the article/**Supplementary Material**. Further inquiries can be directed to the corresponding authors.

## References

[1] Deng H Li M Fang X Zhang J Wang J Tang K . Evaluation of the mechanical properties and clinical application of nickel-titanium shape memory alloy anal fistula clip. Front Surg. (2023) 10:1235666. doi: 10.3389/fsurg.2023.1235666, PMID: 37680263 PMC10481869

[2] Deng H Zhang J Yuan X . The effects of phellodendron decoction on wound healing of anal fistula after anal fistulotomy. Evid Based Complement Alternat Med. (2022) 2022:7363006. doi: 10.1155/2022/7363006, PMID: 36016687 PMC9396417

[3] Zhang W Feng W Chen J Cao R Chen X . Exosomal miR-93-3p targets EIF4EBP1 to regulate macrophage polarization and accelerate wound healing post-anal fistula surgery. Front Pharmacol. (2025) 16:1599633. doi: 10.3389/fphar.2025.1599633, PMID: 40900833 PMC12399553

[4] Brinkmann V Reichard U Goosmann C Fauler B Uhlemann Y Weiss DS . Neutrophil extracellular traps kill bacteria. Science. (2004) 303:1532–5. doi: 10.1126/science.1092385, PMID: 15001782

[5] Zhu Z Zhou S Li S Gong S Zhang Q . Neutrophil extracellular traps in wound healing. Trends Pharmacol Sci. (2024) 45:1033–45. doi: 10.1016/j.tips.2024.09.007, PMID: 39419742

[6] James P Kaushal D Beaumont Wilson R . Netosis in surgery: pathophysiology, prevention and treatment. Ann Surg. (2024) 279:765–80. doi: 10.1097/SLA.0000000000006196, PMID: 38214150 PMC10997183

[7] Papayannopoulos V . Neutrophil extracellular traps in immunity and disease. Nat Rev Immunol. (2018) 18:134–47. doi: 10.1038/nri.2017.105, PMID: 28990587

[8] Elrod J Lenz M Kiwit A Armbrust L Schonfeld L Reinshagen K . Murine scald models characterize the role of neutrophils and neutrophil extracellular traps in severe burns. Front Immunol. (2023) 14:1113948. doi: 10.3389/fimmu.2023.1113948, PMID: 36825027 PMC9941538

[9] Lu W Li X Wang Z Zhao C Li Q Zhang L . Mesenchymal stem cell-derived extracellular vesicles accelerate diabetic wound healing by inhibiting NET-induced ferroptosis of endothelial cells. Int J Biol Sci. (2024) 20:3515–29. doi: 10.7150/ijbs.97150, PMID: 38993565 PMC11234223

[10] Deng H Li M Fang X Tang K Xu S Ding R . Zuoqing granules attenuate ulcerative colitis via macrophage polarization modulation: involvement of the PPAR-gamma/NF-kappaB/STAT1 signaling axis. Front Pharmacol. (2025) 16:1646545. doi: 10.3389/fphar.2025.1646545, PMID: 40860883 PMC12375609

[11] Li M Deng H Xu S Fang X Tang K Chen L . The active ingredients and mechanism of Zuoqing San in the treatment of sigmoid ulcerative colitis by retention enema. J Complement Integr Med. (2025) 22:343–52. doi: 10.1515/jcim-2024-0435, PMID: 39983068

[12] Percie Du Sert N Hurst V Ahluwalia A Alam S Avey MT Baker M . The ARRIVE guidelines 2.0: Updated guidelines for reporting animal research. PloS Biol. (2020) 18:e3000410. doi: 10.1371/journal.pbio.3000410, PMID: 32663219 PMC7360023

[13] Wang L Qi W Gao J Tian M Xu J . Tongyangxiao Lotion promotes postoperative wound healing in a rat model of anal fistula by downregulating inflammatory factors and suppressing inflammation. Nan Fang Yi Ke Da Xue Xue Bao. (2024) 44:1256–65. doi: 10.12122/j.issn.1673-4254.2024.07.05, PMID: 39051071 PMC11270659

[14] Zhu G Gao B Fan J Chen J Su S Yang X . ICG-mediated fluorescence-assisted debridement to promote wound healing. PloS One. (2023) 18:e0291508. doi: 10.1371/journal.pone.0291508, PMID: 37733658 PMC10513195

[15] Sousa P Moreira A Lopes B Sousa AC Coelho A Rema A . Honey, gellan gum, and hyaluronic acid as therapeutic approaches for skin regeneration. Biomedicines. (2025) 13:508. doi: 10.3390/biomedicines13020508, PMID: 40002923 PMC11853393

[16] Li H Xie H Zhang J Tang C Tian S Yuan P . Hydrogel-based sequential photodynamic therapy promotes wound healing by targeting wound infection and inflammation. Nano Lett. (2025) 25:12107–17. doi: 10.1021/acs.nanolett.5c00014, PMID: 40762315

[17] Yanyan X Renjin T Xuelin LI Hong L . Effect of Neibu Huangqi Youhua formula on postoperative wound healing, inflammatory factors and pain mediators of anal fistula. J Tradit Chin Med. (2025) 45:628–32. doi: 10.19852/j.cnki.jtcm.2025.03.015, PMID: 40524301 PMC12134306

[18] Ma X Li J Li M Qi G Wei L Zhang D . Nets in fibrosis: Bridging innate immunity and tissue remodeling. Int Immunopharmacol. (2024) 137:112516. doi: 10.1016/j.intimp.2024.112516, PMID: 38906006

[19] Fantone KM Gokanapudi N Rada B . Neutrophil extracellular traps and interleukin-1beta in cystic fibrosis lung disease. Front Immunol. (2025) 16:1595994. doi: 10.3389/fimmu.2025.1595994, PMID: 40791588 PMC12337492

[20] Kim HJ Lee YS Lee BS Han CH Kim SG Kim CH . NLRP3 inflammasome activation and NETosis positively regulate each other and exacerbate proinflammatory responses: implications of NETosis inhibition for acne skin inflammation treatment. Cell Mol Immunol. (2024) 21:466–78. doi: 10.1038/s41423-024-01137-x, PMID: 38409251 PMC11061142

[21] Xu W Bradstreet TR Zou Z Hickerson S Zhou Y He H . Reprogramming aerobic metabolism mitigates Streptococcus pyogenes tissue damage in a mouse necrotizing skin infection model. Nat Commun. (2025) 16:2559. doi: 10.1038/s41467-025-57348-x, PMID: 40089471 PMC11910614

[22] Wu Y Ning K Huang Z Chen B Chen J Wen Y . NETs-CD44-IL-17A feedback loop drives th17-mediated inflammation in behcet’s uveitis. Adv Sci (Weinh). (2025) 12:e2411524. doi: 10.1002/advs.202411524, PMID: 40013981 PMC12021058

[23] Malamud M Whitehead L Mcintosh A Colella F Roelofs AJ Kusakabe T . Recognition and control of neutrophil extracellular trap formation by MICL. Nature. (2024) 633:442–50. doi: 10.1038/s41586-024-07820-3, PMID: 39143217 PMC11390483

[24] Chen Y Tetz ZA Zeng X Go SJ Ouyang W Lee KE . CitH3, a druggable biomarker for human diseases associated with acute NETosis and chronic immune dysfunction. Pharmaceutics. (2025) 17:809. doi: 10.3390/pharmaceutics17070809, PMID: 40733019 PMC12300630

[25] Wei H Xia D Li L Liang L Ning L Gan C . Baicalin modulates glycolysis via the PKC/raf/MEK/ERK and PI3K/AKT signaling pathways to attenuate IFN-I-induced neutrophil NETosis. Mediators Inflammation. (2025) 2025:8822728. doi: 10.1155/mi/8822728, PMID: 40420943 PMC12105894

[26] Muraro SP Souza De G. F Gallo SW Silva Da B. K . Respiratory Syncytial Virus induces the classical ROS-dependent NETosis through PAD-4 and necroptosis pathways activation. Sci Rep. (2018) 8:14166. doi: 10.1038/s41598-018-32576-y, PMID: 30242250 PMC6154957

[27] Zhuang H Song X Li J Li Z Wu S Wang P . EMAP-II from macrophage-derived extracellular vesicles drives neutrophil extracellular traps formation via PI3K/AKT/mtROS in lung ischemia/reperfusion injury. Redox Biol. (2025) 85:103750. doi: 10.1016/j.redox.2025.103750, PMID: 40616949 PMC12271427

[28] Baratchi S Danish H Chheang C Zhou Y Huang A Lai A . Piezo1 expression in neutrophils regulates shear-induced NETosis. Nat Commun. (2024) 15:7023. doi: 10.1038/s41467-024-51211-1, PMID: 39174529 PMC11341855

[29] Guo G Liu Z Yu J You Y Li M Wang B . Neutrophil function conversion driven by immune switchpoint regulator against diabetes-related biofilm infections. Adv Mater. (2024) 36:e2310320. doi: 10.1002/adma.202310320, PMID: 38035713

[30] Fan L Jia X Dong F Yang S Li W Zhao R . Ginseng-derived nanoparticles accelerate diabetic wound healing by modulating macrophage polarization and restoring endothelial cell function. Mater Today Bio. (2025) 34:102143. doi: 10.1016/j.mtbio.2025.102143, PMID: 40786656 PMC12332952

[31] Huang YF Wang G Ding L Bai ZR Leng Y Tian JW . Lactate-upregulated NADPH-dependent NOX4 expression via HCAR1/PI3K pathway contributes to ROS-induced osteoarthritis chondrocyte damage. Redox Biol. (2023) 67:102867. doi: 10.1016/j.redox.2023.102867, PMID: 37688977 PMC10498433

[32] Liu X Arfman T Wichapong K Reutelingsperger CPM Voorberg J Nicolaes GAF . PAD4 takes charge during neutrophil activation: Impact of PAD4 mediated NET formation on immune-mediated disease. J Thromb Haemost. (2021) 19:1607–17. doi: 10.1111/jth.15313, PMID: 33773016 PMC8360066

