# Correction: Pathological neutrophil extracellular traps hinder postoperative anal fistula wound healing and are attenuated by Zuoqing granule via suppression of the Nox4 pathway

**DOI:** 10.3389/fimmu.2026.1792671

**Published:** 2026-01-28

**Authors:** Xiaoli Fang, Heng Deng, Ming Li, Xiang Gao, Chunrong He, Hui Liu

**Affiliations:** 1 Department of Anorectal Surgery, The First Affiliated Hospital of Anhui University of Chinese Medicine, Hefei, China; 2 Department of Anorectal Surgery, Second Affiliated Hospital, Anhui University of Chinese Medicine, Hefei, China; 3 Department of Anorectal Surgery, The Third Affiliated Hospital of Anhui University of Chinese Medicine, Hefei, China; 4 General Practice Department, Hefei Haiheng Health Service Center, Hefei, China; 5 General Practice Department, Anhui Provincial Second People’s Hospital, Hefei, Anhui, China

**Keywords:** anal fistula, chronic inflammation, NEtosis, NOX4, wound healing, Zuoqing granule

Affiliation “Department of Anorectal Surgery, The First Affiliated Hospital of Anhui University of Chinese Medicine, Hefei, China” and "Department of Anorectal Surgery, The Third Affiliated Hospital of Anhui University of Chinese Medicine, Hefei, China" were omitted for author “Ming Li”. These affiliations have now been added for author “Ming Li”.

Author “Ming Li” was erroneously assigned to affiliation “Department of Anorectal Surgery, Second Affiliated Hospital, Anhui University of Chinese Medicine, Hefei, China”. This affiliation has now been removed for author “Ming Li”.

The original version of this article has been updated.

