# Supplementary material for: Pathological neutrophil extracellular traps hinder postoperative anal fistula wound healing and are attenuated by Zuoqing granule via suppression of the Nox4 pathway
Source: Front Immunol. 2026 Jan 20;16:1730184. doi: 10.3389/fimmu.2025.1730184 (PMC12892496; doi:10.3389/fimmu.2025.1730184)
Supplement: Supplementary file 1 [file Table1.docx]

Table S1. Chemical characterization of Zuoqing Granule by UPLC-MS.

| Serial Number | Identified active compounds | m/z | Rt (min) | Score |
| --- | --- | --- | --- | --- |
| 1 | Sophoridine | 249.1958 | 2.2 | 0.9998 |
| 2 | Oxysophocarpine | 263.1754 | 2.4 | 0.9969 |
| 3 | Oxymatrine | 265.1909 | 2.5 | 0.9949 |
| 4 | 13-Hydroxylupanine | 265.1908 | 2.8 | 0.9838 |
| 5 | L-Tryptophan | 205.0971 | 2.9 | 0.9783 |
| 6 | Ethyl [(diphenylacetyl)amino]acetate | 298.1438 | 3.21 | 0.9634 |
| 7 | Phellodendrine | 342.17 | 3.42 | 0.9996 |
| 8 | Sibiricose A5 | 177.0546 | 3.53 | 0.9881 |
| 9 | Trp-Gly-Trp | 448.1965 | 3.51 | 0.7386 |
| 10 | Magnoflorine | 342.1698 | 3.60 | 0.929 |
| 11 | Codethyline | 314.1748 | 4.02 | 0.915 |
| 12 | 6-O-((2E)-3-(4-Hydroxy-3- methoxyphenyl)prop-2- enoyl)hex-2-ulofuranosyl hexopyranoside | 177.0547 | 4.2 | 0.9884 |
| 13 | 3'-Amino-3,4- dimethylbenzophenone | 120.0446 | 4.7 | 0.9934 |
| 14 | Majarine | 336.1228 | 5.9 | 0.9857 |
| 15 | (+)-Maackiain | 285.0756 | 6.8 | 0.9982 |
| 16 | Kurarinone | 437.197 | 11.51 | 0.9942 |
| 17 | (2R,3S)-Piscidic acid | 255.0509 | 2.5 | 0.7671 |
| 18 | Leiocarposide | 345.0826 | 2.6 | 0.9174 |
| 19 | Cynarin | 353.0878 | 2.8 | 0.9805 |
| 20 | 3-O-Coumaroylquinic acid | 337.093 | 3.31 | 0.991 |
| 21 | (+)-catechin hydrate | 289.0719 | 3.45 | 0.9962 |
| 22 | 5-Feruloylquinic acid | 367.1034 | 3.51 | 0.9939 |
| 23 | 1-Isopropylbenzimidazole-2-sulfonic acid | 239.056 | 3.6 | 0.7961 |
| 24 | Quercetrin | 447.0934 | 5.5 | 0.984 |
| 25 | Azelaic acid | 187.0971 | 6.2 | 0.9985 |
| 26 | (2E)-3,7-Dimethylocta-2,6-dien-1-yl 6-O-.alpha.-Larabinofuranosyl-.beta.-Dglucopyranoside | 493.2291 | 7.72 | 0.8311 |
| 27 | FA 18:2+3o | 327.2177 | 9.1 | 0.9886 |
| 28 | 3-Ara-28-Glu Hederagenin | 811.4488 | 9.6 | 0.9446 |
| 29 | Evodin | 515.1922 | 9.7 | 0.9867 |
| 30 | 9-Octadecenoic acid | 329.2334 | 9.9 | 0.9949 |
| 31 | 5,8,11-trihydroxy-3-Asarylpropionic acid | 239.0923 | 10.3 | 0.8992 |
