# Supplementary figures and images for: Pathological neutrophil extracellular traps hinder postoperative anal fistula wound healing and are attenuated by Zuoqing granule via suppression of the Nox4 pathway
Source: Front Immunol. 2026 Jan 20;16:1730184. doi: 10.3389/fimmu.2025.1730184 (PMC12892496; doi:10.3389/fimmu.2025.1730184)

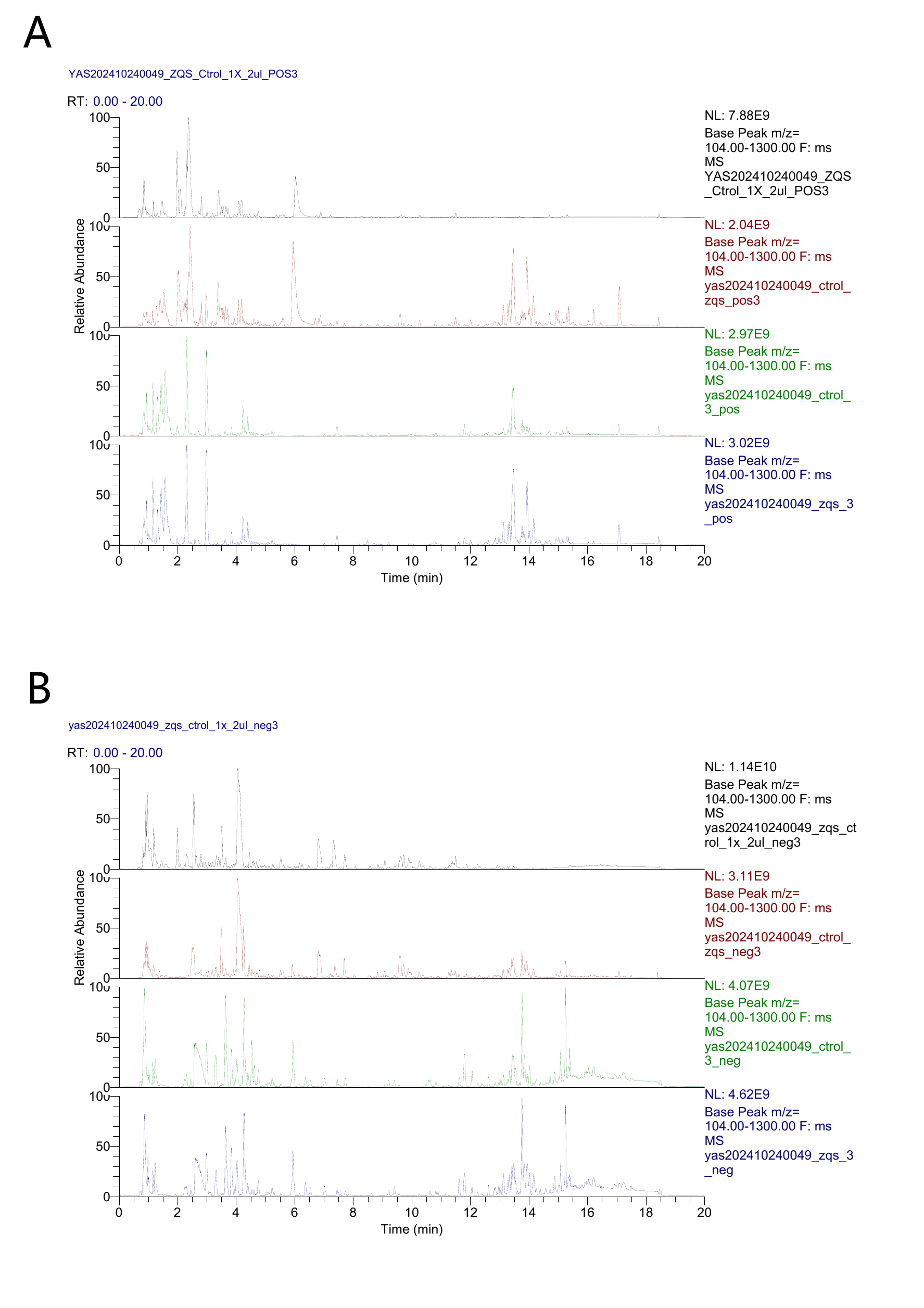

Supplement: Supplementary file 2 [file Image1.png]
